# Trained immunity in lung injury and repair

**DOI:** 10.3389/fimmu.2026.1819744

**Published:** 2026-04-17

**Authors:** Thi Hong Nhung Pham, Bisheng Zhou

**Affiliations:** Department of Pharmacology and Regenerative Medicine, University of Illinois College of Medicine, Chicago, IL, United States

**Keywords:** ARDS, innate immune cells, lung injury and repair, memory, trained immunity

## Abstract

Trained immunity refers to a phenomenon in which innate immune cells undergo long-lasting functional adaptation following an initial challenge. This paradigm shift in pulmonary immunology reveals that classical innate cells such as alveolar macrophages and hematopoietic progenitors, as well as structural cells including epithelial and endothelial populations, can acquire memory-like traits through sustained epigenetic and metabolic reprogramming. In the lung, these reprogrammed circuits influence host defense, lung injury, repair, and fibrotic remodeling through mechanisms involving histone H3K4 trimethylation, mTOR–HIF-1α-dependent metabolic shifts, and KLF4–MERTK-driven efferocytosis. Recent studies highlight the dual nature of this response: whereas appropriately tuned training accelerates pathogen clearance and supports epithelial repair, excessive or persistent activation can exacerbate maladaptive inflammation and fibrosis. In this Mini-Review, we summarize advances from 2020 to 2025 on the cellular and molecular regulation of pulmonary trained immunity, its emerging roles in acute respiratory distress syndrome (ARDS) and lung fibrosis, and novel therapeutic approaches that aim to modulate innate immune reprogramming for lung repair.

## 1 Introduction

For decades, immunological memory was viewed as an exclusive hallmark of the adaptive immune system, driven by antigen-specific T and B lymphocytes. Discoveries over the past decade have challenged this view, showing that innate immune cells can also acquire sustained, memory-like functional characteristics through a process termed trained immunity. This adaptive-like property arises from epigenetic and metabolic reprogramming initiated by microbial or endogenous components, which subsequently shape the magnitude and quality of innate responses upon secondary challenge (1–5).

Experimentally, trained immunity is defined using a characteristic two-step stimulation model. Innate immune cells or their progenitors are first exposed to a primary stimulus (such as β-glucan, BCG, or LPS), followed by a resting phase that allows the resolution of acute activation. Cells are then re-stimulated with either the same or an unrelated secondary stimulus. A trained phenotype is identified by a quantitatively or qualitatively altered response upon re-challenge, including enhanced cytokine production, increased microbial killing, or improved efferocytosis (3, 6, 7). Importantly, the inclusion of a resting period distinguishes trained immunity from transient activation, as it ensures that the observed effects reflect durable cellular reprogramming rather than residual signaling from the initial stimulus. In many systems, additional evidence, such as persistence of epigenetic marks (e.g., H3K4me3) or metabolic rewiring, further supports the presence of a trained state (3–5).

The establishment of trained immunity in pulmonary cells is governed by coordinated epigenetic remodeling, metabolic reprogramming, and transcriptional rewiring. The lung, a mucosal organ continuously exposed to environmental challenges, represents a critical interface for innate immune adaptation. It comprises various distinct cell types that function cooperatively to maintain tissue homeostasis and defense. Among these, alveolar macrophages (AMs), endothelial cells, and epithelial cells play a major role in orchestrating inflammatory and reparative processes. Recent studies have highlighted that these cells, along with bone marrow-derived progenitors, possess the capacity to sustain functional reprogramming long after the initial stimulus has resolved (6–15). Understanding pulmonary-trained immunity provides new insights into the mechanisms linking acute injury to chronic lung disease. By linking cellular diversity with molecular pathways and functional outcomes, this review synthesizes the most recent advances (2020–2025) on how trained immunity influences the onset, progression, and resolution of acute respiratory distress syndrome (ARDS) and pulmonary fibrosis and discusses how its targeted modulation could inform next-generation therapeutic strategies in regenerative medicine.

## 2 Cellular players of pulmonary trained immunity

**Figure 1:**
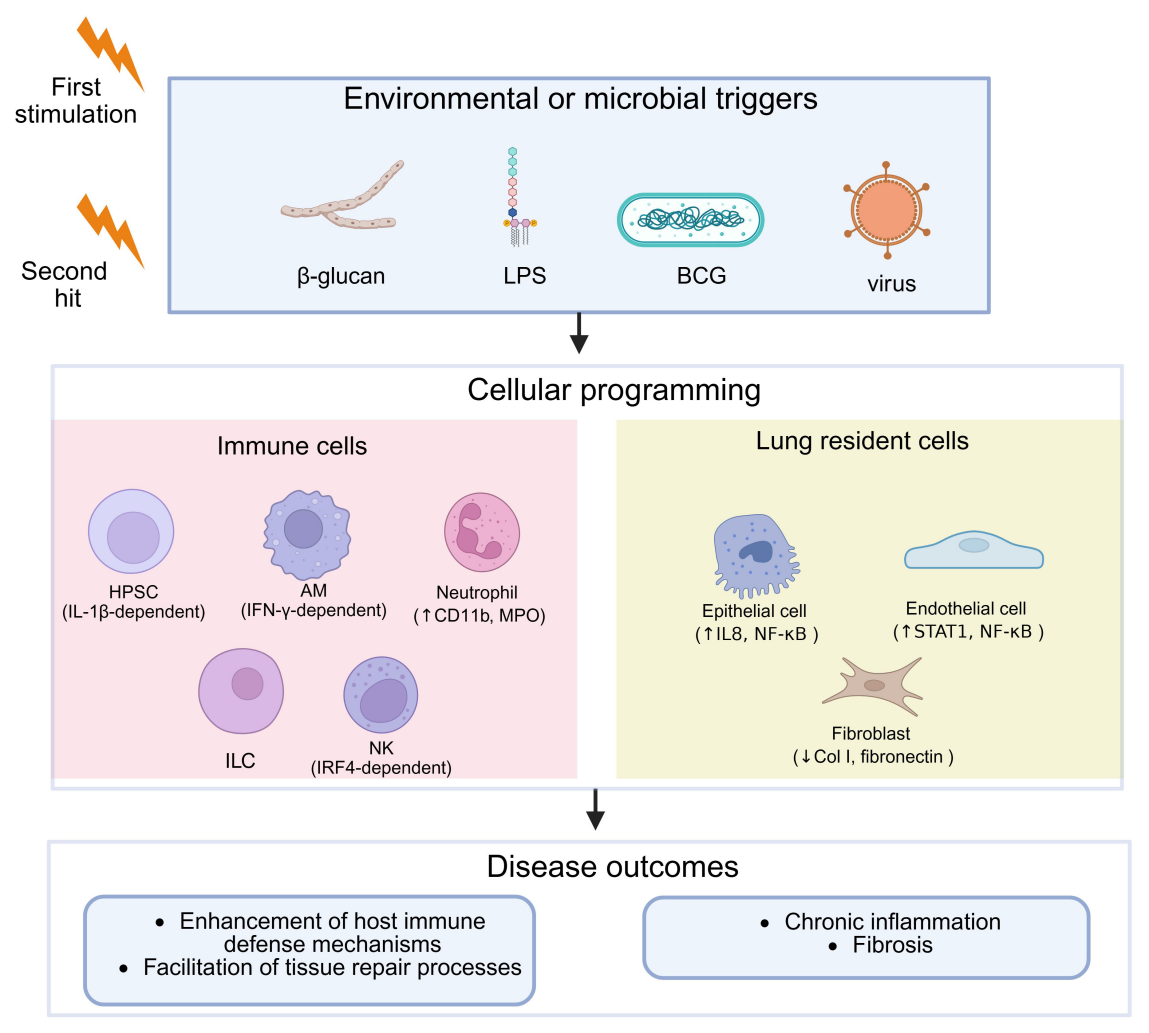
Induction and consequences of trained immunity in the lung.

The lung contains a highly diverse array of immune and structural cells that work in concert to maintain host defense, immune tolerance, and tissue repair. Recent progress in the field has broadened the concept of trained immunity—once considered a feature limited to monocytes and macrophages—to include a wider spectrum of pulmonary cell populations capable of developing memory-like functional states through coordinated epigenetic and metabolic remodeling (6, 8, 16, 17). The following sections synthesize key findings and elucidate the major cellular contributors to pulmonary-trained immunity (**Figure 1**).

### 2.1 Alveolar macrophages: long-lived sentinels with epigenetic and functional memory

Alveolar macrophages (AMs), positioned at the alveolar interface, are the principal sentinels of the lower respiratory tract. Traditionally viewed as terminally differentiated phagocytes, AMs are now recognized as self-renewing and capable of sustaining epigenetic memory following primary immune activation. Recent evidence demonstrates that AMs undergo chromatin remodeling and metabolic rewiring in response to microbial or inflammatory factors, thereby establishing an innate memory state (18–22). Previous studies reveal that respiratory viral infections such as influenza A virus (IAV) or SARS-CoV-2 prime AMs to adopt a trained phenotype characterized by augmented MHC class II expression, enrichment of type I interferon-associated transcriptional programs, and enhanced capacity for pathogen clearance upon secondary challenge. The induction of these reprogrammed AMs requires IFN-γ-dependent signaling from CD8^+^ T cells, highlighting a mechanistic interface between adaptive and innate immunity (20). Interestingly, these trained AMs persist independently of bone marrow replenishment under homeostasis, indicating local self-renewal as a mechanism for the maintenance of innate immune memory (20, 21).

Distinct microbial ligands can elicit a diverse range of outcomes. For example, repeated exposure to low-dose intranasal lipopolysaccharide (LPS) induces a protective role against Streptococcus pneumoniae, whereas β-glucan-mediated stimulation enhances efferocytosis and mitigates fibrosis in bleomycin-injured lungs through KLF4-MERTK-dependent pathways (19, 23). In addition, systemic Bacillus Calmette-Guérin (BCG) vaccination confers heterologous protection by modulating the gut-lung axis, wherein microbial metabolites translocate to the lung and augment macrophage transcriptional programming via mTORC2/HK1-dependent metabolic pathway (4, 17, 24, 25). Collectively, these findings demonstrate that AMs are autonomous effectors of pulmonary trained immunity, which can mediate both protective and maladaptive inflammation.

### 2.2 Hematopoietic stem and progenitor cells: central reservoirs of long-term innate memory

In lung injury, the persistence of trained immunity cannot be explained solely by tissue-resident macrophage self-renewal. Instead, long-term reprogramming frequently originates in the bone marrow, where HSPCs undergo epigenetic imprinting that shapes the phenotype of circulating monocytes subsequently recruited to the injured lung. Because mature myeloid cells are short-lived, the persistence of trained immunity is frequently rooted in the reprogramming of hematopoietic stem and progenitor cells (HSPCs) within the bone marrow (6, 26). Exposure to systemic agonists, such as β-glucan or BCG, initiates chromatin remodeling at enhancer and promoter regions in HSPCs, resulting in a long-lasting transcriptional bias toward myelopoiesis and an augmented inflammatory state. The progeny of these reprogrammed progenitors exhibits heightened responsiveness and contribute to sustained immune adaptation in the lung (6, 7, 26).

Notably, recent research in murine models of systemic lupus erythematosus (SLE) has shown that HSPCs acquire disease-associated transcriptional imprints that can be transferred to naïve recipients via bone marrow transplantation. Bone marrow-derived macrophages exhibit enhanced cytokine production and bactericidal activity despite reduced glycolytic flux, suggesting the coexistence of distinct metabolic and functional states. Similarly, post-stroke HSPCs undergo IL-1β-dependent transcriptional remodeling that drives the development of chronic inflammatory monocyte phenotypes, which, upon migration to peripheral organs such as the lungs, exacerbate tissue injury (27–31). These findings establish the bone marrow as a central hub for generating innate immune memory, which can shape pulmonary responses. Although these findings arise from systemic injury models, they illustrate how HSPC imprinting can generate monocytes with durable inflammatory biases that influence peripheral organs, including the lung during ARDS or fibrotic remodeling.

### 2.3 Additional immune contributors: neutrophils, natural killer cells, and innate lymphoid cells

Although trained immunity is classically defined by durable epigenetic reprogramming in long-lived innate cells or their progenitors, emerging evidence suggests that certain short-lived innate populations can exhibit functional reprogramming following prior stimulation. In humans, neutrophils isolated from BCG-vaccinated individuals display enhanced oxidative burst, increased expression of activation markers (CD11b, MPO), and augmented cytokine production several months post-vaccination. Given the limited lifespan of circulating neutrophils, these persistent changes likely reflect epigenetic remodeling in granulocyte–monocyte progenitors in the bone marrow rather than the intrinsic long-term reprogramming of mature neutrophils. Similarly, β-glucan exposure alters neutrophil trafficking dynamics and enhances efferocytosis within the lung, suggesting that progenitor imprinting can modulate neutrophil-mediated resolution of secondary inflammatory injury (7, 16).

Natural killer (NK) cells represent a unique intersection between innate and adaptive immunity. Following viral infection, NK cells undergo IRF4-dependent chromatin remodeling at effector loci, resulting in increased accessibility at IFN-γ–associated regulatory regions and enhanced recall responses upon re-exposure (32). This epigenetically primed chromatin configuration supports sustained antiviral responsiveness, although whether this phenomenon fulfills strict criteria for trained immunity remains debated.

Likewise, IL-33–experienced group 2 innate lymphoid cells (ILC2s) exhibit sustained histone acetylation at type 2 cytokine loci, maintaining a poised chromatin state that enables rapid cytokine production during subsequent allergen challenge (33–35). However, the durability and progenitor involvement underlying ILC2 imprinting are less well defined.

Collectively, these findings suggest that epigenetic priming extends across multiple innate immune populations. Nevertheless, the extent to which short-lived cells themselves are “trained,” versus reflecting upstream progenitor reprogramming or transient tissue adaptation, remains an important conceptual boundary in defining trained immunity in the lung.

### 2.4 Non-immune structural cells: epithelial, endothelial, and fibroblast memory

Recent research has revealed that non-hematopoietic cells in the lung also possess the capacity for innate immune memory. Human bronchial epithelial cells pre-exposed to Pseudomonas aeruginosa flagellin exhibit enhanced IL-8 secretion and NF-κB activation upon subsequent challenge, a process associated with acetylation of H3K4 and H3K27 at proinflammatory promoters. Alveolar type II (AT2) epithelial cells demonstrate upregulated MHC class II and interferon-responsive gene networks during viral infection, suggesting an antigen-presenting and memory-like role within the epithelial compartment (11, 12, 36).

Pulmonary endothelial cells, which regulate vascular homeostasis and leukocyte trafficking, also exhibit features consistent with trained immunity. Stimulation with oxidized low-density lipoprotein (LDL) or β-glucan induces sustained expression of ICAM-1, VCAM-1, IL-6, and MCP-1 through prolonged NF-κB and STAT1 signaling, reflecting persistent inflammatory reprogramming. In models of lung injury and fibrosis, single-cell transcriptomic analyses further reveal activated endothelial states characterized by enhanced chemokine production, stress-response programs, and increased expression of pattern-recognition receptors. These findings suggest that endothelial cells can acquire durable immune-like transcriptional programs that modulate leukocyte recruitment and influence fibrotic progression (13, 14, 37–40).

Finally, fibroblasts are central regulators of lung repair and fibrosis. Beyond their structural role in extracellular matrix production, fibroblasts exhibit marked transcriptional plasticity in response to inflammatory and injury-associated cues. Distinct fibroblast subsets contribute either to regenerative repair or to persistent fibrotic remodeling, and sustained activation of myofibroblasts underlies progressive disease such as idiopathic pulmonary fibrosis. Although direct evidence for classical trained immunity in fibroblasts remains limited, their capacity to integrate immune-derived signals into durable functional states suggests that fibroblast–immune crosstalk may critically shape the balance between repair and fibrosis (15). Notably, unlike classical trained immunity in myeloid cells and their progenitors, which is supported by robust experimental frameworks, including restimulation assays and lineage tracing, evidence for durable, self-sustaining memory states in structural lung cells remains limited. As such, these findings are more appropriately framed as “memory-like” or “primed” states rather than fully established trained immunity.

Together, these findings highlight that innate immune memory is a multicellular process that extends beyond classical immune populations and relies on coordinated interactions between parenchymal and immune cells.

## 3 Molecular mechanisms and functional outcomes

Building upon this framework, trained immunity in pulmonary cells is established through interconnected epigenetic remodeling and metabolic reprogramming that together shape downstream transcriptional responsiveness. These molecular adaptations generate durable functional states capable of mounting rapid and amplified responses upon secondary stimulation (**Table 1**).

**Table 1:** Summary of molecular mechanisms driving pulmonary trained immunity.

Mechanistic axis	Representative molecular changes	Primary cell type(s)	Species/model system	Functional outcome	Key reference
Histone modifications	↑H3K4me3, ↑H3K27ac, ↓H3K9me2	AMs, HSPCs	Murine models; human BCG vaccination studies	Rapid cytokine induction and transcriptional priming	(3, 14)
mTOR–HIF-1α pathway	Enhanced glycolysis, fumarate accumulation	AMs	Human monocytes; murine macrophage models	Reinforced epigenetic activation and metabolic reprogramming	(4)
KLF4–MERTK signaling	Upregulation of efferocytosis genes	AMs	Murine bleomycin-induced lung injury model	Resolution of injury and enhanced efferocytosis	(19)
Mevalonate metabolism	Cholesterol and lipid biosynthesis	Endothelial cells, epithelial cells	Human and murine endothelial cell studies	Increased cytokine and endothelial activation	(12)
IL-1β–dependent axis	Epigenetic imprinting of HSPCs	BM-derived macrophages	Murine systemic inflammatory injury models	Systemic inflammatory memory	(30)
Gut–lung axis	NOD2-mediated cross-organ signaling	AMs, epithelial cells	Murine BCG vaccination model	Enhanced mucosal defense and macrophage training	(17)

### 3.1 Epigenetic remodeling

Epigenetic reprogramming constitutes the foundational mechanism underlying trained immunity. Stimulation with microbial or metabolic ligands induces persistent modifications in histone methylation and acetylation, resulting in the establishment of accessible chromatin landscapes at promoters and enhancers of immune-response genes. In alveolar macrophages and bone marrow progenitors, exposure to β-glucan or BCG vaccination enhances trimethylation of histone H3 at lysine 4 (H3K4me3) and acetylation at lysine 27 (H3K27ac), while simultaneously reducing histone H3 at lysine 9 methylation (H3K9me2) (3, 4, 6, 7, 14, 18–20). This chromatin state primes inflammatory and antimicrobial gene loci for rapid transcription upon secondary stimulation.

Endothelial cells trained with oxidized LDL or β-glucan exhibit similar epigenetic hallmarks, including increased histone H3 lysine 14 acetylation (H3K14ac) and sustained occupancy of NF-κB at promoter regions of cytokine genes (IL6, ICAM1, MCP1) (2, 5, 13, 14). Moreover, long non-coding RNAs (lncRNAs) and DNA methylation dynamics have also emerged as critical modulators of trained immunity, contributing to the transcriptional persistence of inflammatory and reparative programs (3–5, 14, 26). These modifications collectively establish a transcriptionally permissive chromatin state that underpins the longevity of innate memory within the pulmonary environment.

### 3.2 Metabolic rewiring

Metabolic reprogramming functions as both an initiator and effector of trained immunity. The metabolic shift from oxidative phosphorylation (OXPHOS) to aerobic glycolysis, driven by the mTOR-HIF-1α axis, facilitates rapid ATP generation and provides intermediates for histone-modifying enzymes. β-glucan–trained macrophages exhibit elevated glycolytic flux and fumarate accumulation, which inhibits KDM5 histone demethylases, reinforcing H3K4me3 enrichment. Parallel increases in cholesterol synthesis, fatty acid desaturation, and glutaminolysis sustain redox homeostasis and promote cytokine biosynthesis (3–5, 14, 24, 26).

Distinct metabolic trajectories determine functional outcomes: while glycolytic dominance supports proinflammatory responsiveness, enhanced fatty acid oxidation and mitochondrial biogenesis favor reparative phenotypes, such as the KLF4–MERTK–dependent efferocytosis observed in β-glucan–trained alveolar macrophages (3–5, 14, 18, 19, 24, 26, 41). In epithelial and endothelial cells, metabolites of the mevalonate pathway act as secondary messengers that reinforce histone acetylation and NF-κB activation (2, 5, 13, 14). Thus, metabolic circuits serve as key molecular intermediaries linking environmental cues to epigenetic remodeling and gene expression reprogramming.

### 3.3 Transcriptional and signaling networks

Trained immunity involves the integration of multiple signaling pathways and transcription factor modules. Upstream pattern-recognition receptors—including Toll-like receptors (TLRs) and NOD-like receptors such as NOD2—activate convergent NF-κB and MAPK signaling pathways that translate microbial stimulation into sustained transcriptional programs (3, 4, 14, 23). Downstream, transcription factors including KLF4, STAT1, IRF family members, and C/EBPβ contribute to gene-specific activation programs that shape effector phenotypes. KLF4 promotes macrophage efferocytosis by upregulating MERTK in trained alveolar macrophages, thereby enhancing injury resolution. In antiviral contexts, type I interferon–dependent signaling involving STAT1 and IRF9 is associated with memory-like responses following SARS-CoV-2 infection (18, 19, 22, 41). Epigenetic regulators such as ATF7 modulate the balance between inflammatory amplification and tolerance, underscoring the context-dependent nature of trained immune states (3–5).

Recent data highlight ion flux as an integral component of pulmonary trained immunity. Thompson et al. demonstrated that extracellular ATP could train AMs via P2X7 signaling to promote translocation of the two-pore-domain K+ channel TWIK2 from endosomal compartments to the plasma membrane. TWIK2-mediated K+ efflux activates the NLRP3 inflammasome and reprograms cellular metabolism. Subsequently, the internalization of TWIK2 into the phagosomal membrane enables K+ influx, which optimizes the phagosomal pH and ionic strength for bacterial killing. These findings position ATP–TWIK2–dependent K+ flux as a dual-site regulator of trained AM function, coupling damage-associated molecular patterns (DAMPs) sensing to both inflammasome activation and enhanced microbicidal capacity in the lung (42).

Moreover, inter-organ communication contributes to systemic training. The gut–lung axis, mediated by microbial metabolites including short-chain fatty acids, promotes the formation of trained alveolar macrophages and enhances local immune responsiveness (17, 24). IL-1β–dependent signaling in hematopoietic stem and progenitor cells has been shown to mediate central trained immunity in the bone marrow, illustrating how cytokine networks sustain long-term innate memory while shaping peripheral immune responses (6, 7, 27).

## 4 Clinical relevance: ARDS and lung fibrosis

### 4.1 Trained immunity in acute respiratory distress syndrome

ARDS is characterized by diffuse alveolar damage, impaired gas exchange, and a high mortality rate, typically arising from dysregulated inflammation triggered by infection (e.g., pneumonia, sepsis) or sterile insults (e.g., trauma, pancreatitis). Massive recruitment of monocytes and neutrophils into the alveolar compartment and their activation in response to pathogen-associated molecular patterns (PAMPs) and DAMPs drive a cytokine storm, excess production of reactive oxygen species (ROS), and epithelial-endothelial barrier injury (9, 43).

Experimental models suggest that AMs are central mediators of trained responses in ARDS. Repetitive low-dose LPS exposure induces a reprogrammed AM phenotype characterized by the upregulation of anti-apoptotic genes (MCL1) and the downregulation of pro-apoptotic genes (BAX). This shift promotes macrophage survival within the injured alveolar niche, preserves their efferocytic capacity, and supports restoration of the epithelial–endothelial barrier. By sustaining a pool of competent AMs that efficiently clear apoptotic cells and debris, trained immunity can limit the amplification of inflammation and facilitate more orderly resolution of acute lung injury (18, 19).

Beyond macrophages, trained immunity might also modulate neutrophil behavior in ARDS resolution. β-glucan-induced training reprograms neutrophil inflammatory responses, promoting antimicrobial activity while restraining excessive inflammation and ROS, thereby potentially mitigating collateral tissue damage (2, 4, 16). Importantly, these effects differ fundamentally from pharmacologic immunosuppression: trained immunity seeks to restore an appropriate magnitude and quality of innate responses rather than simply suppressing them, thereby reducing the risk of post-ARDS complications such as secondary bacterial pneumonia (3, 5, 22, 26, 31).

Recent translational work further supports the therapeutic potential of trained immunity–based interventions in ARDS. Systemic β-glucan administration in preclinical models reprograms bone marrow progenitors and circulating monocytes through pattern-recognition pathways (including Dectin-1- and TLR2–NF-κB-dependent signaling), leading to dampened systemic cytokine release, improved hemodynamic stability, and enhanced survival (2, 4, 44). Collectively, these findings suggest that judicious induction or modulation of trained immunity may offer a strategy to shift pulmonary innate responses away from injurious hyperinflammation and toward efficient pathogen clearance and tissue repair, while maintaining essential host defense.

### 4.2 Trained immunity in lung fibrosis

Pulmonary fibrosis (PF), typified by irreversible scarring and architectural distortion, represents a maladaptive outcome of unresolved inflammation. Recurrent epithelial injury, dysregulated macrophage activation, and fibroblast proliferation drives extracellular matrix (ECM) accumulation (8, 10, 15, 45). Within this milieu, trained immunity can be either protective or pathogenic, depending on the initiating stimulus and local metabolic programming, shaping repair versus fibrotic trajectories (26–30). Experimental studies using murine models of bleomycin-induced injury have demonstrated that β-glucan–mediated training significantly attenuates lung damage through coordinated macrophage and epithelial responses. In these models, trained alveolar macrophages exhibit elevated expression of KLF4 and its downstream target MERTK, facilitating efficient efferocytosis and clearance of apoptotic cells (19, 41). This process correlates with the induction of epithelial SIRT1 expression, which confers cytoprotective effects by limiting apoptosis and preserving barrier integrity (8, 41).

In preclinical studies, systemic β-glucan pretreatment has been shown to mitigate the severity of fibrosis in bleomycin-challenged mice, resulting in reduced collagen deposition and mortality. The protective effect is mechanistically linked to trained macrophages exhibiting augmented efferocytosis, production of pro-resolving mediators such as Resolvin D1, and suppression of profibrotic cytokines (TGF-β, IL-13). Concurrently, epithelial cells exposed to signals from trained macrophages upregulate SIRT1 and other cytoprotective pathways, thereby limiting epithelial–mesenchymal transition (EMT) and tissue scarring (8, 19, 41).

Conversely, chronic or dysregulated trained responses can exacerbate fibrosis (5, 26, 28–30). Persistent activation of alveolar macrophages through IL-1β–driven signaling in trained hematopoietic progenitors sustains myeloid infiltration and inflammatory cytokine production, fostering fibroblast activation (6, 7, 10, 15, 27, 30). Endothelial cells also contribute through the sustained release of NF-κB–mediated cytokines and the endothelial-to-mesenchymal transition (EndoMT), thereby amplifying ECM deposition (13, 14, 37–40). These observations highlight the dual-edged nature of trained immunity; wherein beneficial tissue repair can progress to pathological remodeling if regulatory checkpoints are compromised.

Emerging single-cell analyses from 2024 to 2025 have further delineated the contribution of endothelial plasticity to fibrosis progression, revealing persistent, injury-trained endothelial states (37, 40). Post-injury endothelial cells exhibit FOXF1/R-Ras dysregulation, perpetuating fibroblast activation and collagen deposition (39, 40). Targeting these maladaptive trained programs through IL-1β blockade, mTOR inhibition, or modulation of metabolic flux has therefore emerged as a promising antifibrotic strategy (4, 5, 26).

## 5 Therapeutic perspectives and future directions

### 5.1 Vaccine-based modulation of innate memory

The BCG vaccine exemplifies microbial induction of trained immunity, providing broad, non-specific protection through hematopoietic and tissue-resident reprogramming (3, 6, 7). Intravenous or mucosal BCG elicits lung-resident macrophages with enhanced antimicrobial activity via the mTORC2–HK1 axis (17, 25). Mucosal delivery platforms, including intranasal formulations, are particularly effective in inducing localized training within alveolar macrophages and epithelial cells, reinforcing barrier integrity (11, 12, 17, 25).

A particularly illustrative example is provided by the study of Kaufmann et al., who demonstrated that intravenous BCG vaccination reprograms long-term hematopoietic stem cells (HSCs) in the bone marrow, rather than merely circulating monocytes. Using bone marrow transplantation experiments in mice, the authors showed that HSCs isolated from BCG-treated donors conferred enhanced protection against Mycobacterium tuberculosis infection when transferred into naïve recipients. Mechanistically, this central training required IL-1β signaling and was associated with durable epigenetic remodeling of HSCs, including increased chromatin accessibility at myeloid lineage genes and enrichment of inflammatory transcription factor motifs. Importantly, this reprogramming skewed hematopoiesis toward myelopoiesis without inducing overt stem-cell exhaustion, highlighting a finely tuned balance between enhanced host defense and preservation of regenerative capacity. While the murine model relied on intravenous BCG administration, subsequent human studies have demonstrated transcriptional rewiring of CD34^+^ progenitors following intradermal BCG vaccination, suggesting that similar central training mechanisms may operate in humans, albeit with potentially different magnitudes and route-dependent distributions (6, 7). These findings underscore how vaccine-induced trained immunity can operate at the level of stem-cell fate decisions, providing durable systemic effects beyond short-lived innate effector populations.

### 5.2 Metabolic and epigenetic reprogramming as therapeutic targets

Metabolic and epigenetic plasticity of trained immunity offers opportunities for therapeutic modulation. Inhibition of the mTOR–HIF-1α axis, a central driver of glycolytic rewiring, can dampen the metabolic programs required for sustained inflammatory training (4). Similarly, β-glucan–based approaches have been shown to reprogram myeloid cells and restore responsiveness in models of sepsis-associated immunoparalysis through TLR2–NF-κB–dependent pathways (44). Because trained immunity depends on persistent chromatin accessibility at inflammatory loci, targeting histone-modifying enzymes represents a potential strategy to restrain maladaptive training states (4). In the pulmonary setting, pro-resolving lipid mediators such as resolvin D1 promote efferocytosis and induce epithelial SIRT1 expression, thereby limiting tissue injury and fibrosis (41). Collectively, these findings indicate that trained immunity represents a dynamically regulated state that can be therapeutically modulated toward either inflammatory amplification or tissue protection. However, translating these approaches into clinical settings will require precise control over the magnitude and duration of immune reprogramming to avoid unintended exacerbation of inflammation or tissue injury.

## 6 Outlook and challenges

Despite substantial progress, several conceptual and practical challenges must be addressed before trained immunity can be safely and effectively harnessed in lung disease. First, the context dependence of trained responses remains poorly defined. The same stimulus (e.g., β-glucan, LPS, viral infection) can induce protective, resolution-biased programs in one setting and maladaptive, profibrotic or hyperinflammatory responses in another. Dissecting how cell type, dose, timing, route of exposure, and host factors such as age, comorbidities, and microbiome composition jointly determine the trajectory of pulmonary trained immunity is essential for rational therapeutic design.

Second, there is an urgent need for robust biomarkers that report on trained states in humans. Most experimental work has relied on ex vivo restimulation assays and bulk or single-cell omics in blood or murine tissues. Translating these insights into clinically useful tools will require scalable assays in blood, bronchoalveolar lavage, or exhaled biomarkers that can distinguish between protective and harmful innate memory, predict the risk of ARDS or fibrosis, and guide the timing of interventions that modulate trained immunity. In addition, an important limitation of the current field is that much of the evidence for trained immunity derives from *in vitro* systems and murine models. Although these studies have defined key mechanisms, they do not fully recapitulate human lung biology. Differences in immune composition and environmental context may influence trained responses, highlighting the need for validation in human tissues and clinically relevant models.

Third, cellular and spatial resolution in the human lung remains limited. While single-cell and spatial transcriptomic approaches are beginning to map the states of AM, interstitial macrophages, endothelial cells, epithelial cells, and fibroblasts in ARDS and fibrotic lungs, it remains unclear which subsets truly embody “trained” programs versus transient activation or exhaustion. Integrating fate-mapping in animal models with high-dimensional profiling of human samples across the continuum from health to injury and repair will be crucial for assigning causality to specific trained cell populations.

Finally, another area that warrants further investigation is the potential role of trained immunity in interstitial lung diseases (ILDs) associated with autoimmune disorders. Although ILD develops only in a subset of patients with these conditions, chronic inflammatory signaling and sustained activation of innate immune pathways may promote long-lasting epigenetic and metabolic reprogramming in myeloid cells and their progenitors. Such persistent innate immune states could contribute to aberrant macrophage activation, endothelial dysfunction, and fibroblast stimulation in the lung microenvironment, thereby influencing fibrotic progression. Exploring whether autoimmune-associated ILD reflects maladaptive trained immunity may therefore provide new insights into disease heterogeneity and identify potential therapeutic targets for modulating innate immune responses in fibrotic lung disease.

Addressing these challenges presents an opportunity not only to clarify the role of innate immune memory in ARDS and lung fibrosis but also to move toward precision therapies that intentionally reprogram innate immune responses. If successful, such strategies could shift clinical practice from merely limiting damage during acute episodes to proactively shaping pulmonary resilience and long-term repair.

## 7 Conclusion

The recognition of trained immunity as a fundamental component of pulmonary host defense and tissue repair has significantly reshaped our understanding of lung injury and repair. Rather than a one-time, stimulus-bound response, innate immunity in the lung is now understood as a dynamic, memory-bearing system in which prior infections, environmental exposures, and tissue damage imprint long-lasting programs on alveolar macrophages, hematopoietic progenitors, and structural cells. These programs can recalibrate the magnitude, quality, and timing of subsequent responses, thereby influencing whether an acute insult resolves with restored architecture or progresses toward chronic inflammation and fibrosis.

Within this framework, ARDS and lung fibrosis can be viewed not simply as consequences of overwhelming injury but as maladaptive outcomes of cellular memory. Appropriately tuned trained immunity can accelerate pathogen clearance, enhance efferocytosis, stabilize epithelial and endothelial barriers, and promote resolution, thereby limiting the transition from acute injury to irreversible scarring. Conversely, when training is driven by persistent cytokine signaling, metabolic stress, or repetitive exposure to PAMPs and DAMPs, it can lock myeloid and structural cells into profibrotic and proinflammatory states that perpetuate damage long after the initial trigger has subsided. In addition to environmental and inflammatory cues, genetic factors may also influence the stability and regulation of trained immune responses. Because trained immunity relies on coordinated epigenetic remodeling, cytokine signaling, and transcriptional regulation, inherited mutations affecting chromatin-modifying enzymes, cytokine receptors, or downstream transcription factors could potentially disrupt the establishment or maintenance of innate immune memory. Investigating inborn errors of immunity affecting these pathways may therefore provide new insights into the molecular determinants of trained immunity and how defects in innate immune memory influence susceptibility to infection, inflammation, or fibrotic remodeling in the lung.

Looking ahead, the concept of trained immunity offers a conceptual and practical bridge between basic mechanistic studies and next-generation therapies. Ultimately, a deeper understanding of trained immunity in the lung has the potential to inform precision immunotherapies that not only treat acute episodes of injury but also promote durable lung repair and resilience.

**Figure 1** schematically summarizes the interplay between stimuli, cellular reprogramming, and disease outcomes in pulmonary trained immunity. Environmental or microbial triggers (β-glucan, BCG, LPS, viral infection) activate both immune and non-immune resident cells, establishing long-lived transcriptional programs via epigenetic and metabolic circuits. The resultant trained phenotypes can enhance host defense and promote repair (e.g., efferocytosis, epithelial protection) or, when dysregulated, drive chronic inflammation and fibrosis. Created with BioRender.com.
